# Diversity engagement is associated with lower burnout among anesthesia providers

**DOI:** 10.1016/j.jcadva.2024.100027

**Published:** 2024-07-17

**Authors:** Julia C. Whiteleather, Beda Rosario-Rivera, Aminat Haruna, Alejandro Munoz-Valencia, Kristin Ondecko-Ligda, Keith M. Vogt, Andrea J. Ibarra

**Affiliations:** a Department of Obstetrics, Gynecology and Reproductive Sciences, Pittsburgh, PA, USA; b Department of Anesthesiology and Perioperative Medicine University of Pittsburgh, Pittsburgh, PA, USA

**Keywords:** Engagement, Diversity, Burnout, Stress, Healthcare providers, Anesthesiology

## Abstract

**Study objective:**

The high prevalence of burnout among anesthesia providers is a well-established, multifactorial problem that deserves systematic attention and action. This study aimed to identify how perceptions of institutional diversity engagement are associated with burnout and perceived stress among anesthesia personnel.

**Design:**

Survey-based prospective cross-sectional study.

**Setting:**

Anesthesiology department that encompasses community and academic hospitals in a large healthcare system.

**Participants:**

One-hundred and sixty anesthesiology department employees over 18 years of age (i.e. attending physicians, trainees, advanced practice providers and others).

**Measurements:**

A web-based survey with 39 questions measured: demographics, diversity engagement, burnout, and perceived stress. The primary objective was to assess the association of burnout and diversity engagement and the mediating effect of perceived stress in this relationship. Our secondary objective was to measure the prevalence of burnout, diversity engagement, and perceived stress. Burnout, diversity engagement, and perceived stress were measured using a validated two-item survey developed from the Maslach Burnout Inventory-Human Services Survey, the 22-item Diversity Engagement Survey (DES), and the Perceived Stress Scale (PSS-4), respectively.

**Main results:**

Mean scores were 4.4 (SD 3.2) for burnout, 78.3 (SD 14.3) for DES, and 4.8 (SD 2.6) for perceived stress. Higher DES score predicted lower burnout (β = −0.11 [95% CI −0.14, −0.08], *P* < 0.001) and lower perceived stress (β = −0.05 [95% CI −0.08, −0.03], *P* < 0.001). Mediation analysis estimated the total effect of burnout (β = −0.10, *P* < 0.001), which comprised the direct effect of diversity engagement (β = −0.08, P < 0.001) and indirect effect of perceived stress (β = −0.02, *P* = 0.0048).

**Conclusions:**

The perception of increased institutional diversity engagement is associated with reduced burnout among anesthesia providers, in part due to a reduction in perceived stress. Implementing interventions at the leadership level that improve diversity engagement may reduce the negative effects of perceived stress and burnout, potentially improving patient care.

## 1. Introduction

Burnout is a prevalent occupational phenomenon characterized by emotional exhaustion, increased mental distance from one’s job, and a sense of reduced personal accomplishment [1–3]. Anesthesia providers exhibit a notably high prevalence of burnout compared to clinicians in other specialties [4–6]. Burnout has been associated with increased medication errors, significant deviation from best practices [7], and lower quality of patient care and safety [8,9]. Workplace factors (e.g. lack of organizational support) also have been associated with an increased likelihood of burnout [10–13]. These findings have prompted a shift towards enhancing workplace culture to better support healthcare providers [8,14].

Diversity can be referred to as the presence of differences within a given setting. These differences can be based on demographic (i.e. race/ethnicity, gender, age), non-demographic (i.e. environmental factors) or social factors (i.e. family size). In the past, institutions have focused on creating metrics to identify differences based on demographic groups, however there is paucity of data measuring how all members are included and engaged. Diversity engagement, a concept rooted in workforce engagement theory, reflects employees’ connection with their institution based on its commitment to diversity and inclusion. Thus, it is defined as the active involvement and commitment of employees in achieving the goals of an institution, fostered by a supportive and inclusive work environment. Recognizing the value of healthcare providers through diversity engagement enhances employee retention and performance [15]. Furthermore, a pro-diversity culture, with aligned perceptions between employees and managers’ perceptions of that culture, fosters an environment that is more conducive to improved individual and overall organizational performance [16].

Using the validated Diversity Engagement Survey [17], we aimed to measure the employee diversity, inclusion dynamics, and organizational culture of a large anesthesiology department across different hospitals. Although burnout results from occupational stress [1,3] and workplace factors may increase susceptibility to burnout and stress [10,18], whether diversity engagement influences burnout and perceived stress among anesthesia providers remains unclear. We hypothesized that higher diversity engagement correlates with lower burnout and perceived stress. Since literature has demonstrated associations between stress and DEI engagement [19,20], as well as stress and burnout [1,2], we expected stress to mediate the diversity engagement-burnout association. Our study provides insights into the relationship between diversity engagement, burnout, and perceived stress, offering a foundation for future interventions aimed at cultivating a more inclusive work environment in anesthesiology departments.

## 2. Methods

This study received University of Pittsburgh Institutional Review Board approval (STUDY22090139). It adhered to the Strengthening the Reporting of Observational Studies in Epidemiology (STROBE) guidelines for reporting observational studies.

### 2.1. Study population and design

This prospective cross-sectional survey study was administered to anesthesia healthcare providers from both community and academic hospitals within University of Pittsburgh Medical Center (UPMC), a multi-site healthcare system based in Western Pennsylvania. Eligible participants were over 18 years of age working as employees (i.e. attending physicians, trainees [residents or fellows], advanced practice providers (APPs) [certified registered nurse anesthetists, nurse practitioners, physician assistants], and Others [anesthesiology technicians, other staff]) in a large UPMC anesthesiology department. A web-based survey was sent out to all eligible participants via email on March 9, 2023. Eligible participants received an email with a link to REDCap where they could electronically sign an informed consent form, review inclusion and exclusion criteria, and complete surveys. Only one response per person was permitted. Two reminder e-mails were sent on March 23, 2023, and April 21, 2023. The survey link was officially closed on April 26, 2023. De-identified data from the surveys were collected; however, participants could submit their e-mail addresses after completing the survey to receive either a $20 Starbucks gift card or one $250 VISA gift card by random selection.

The survey comprised 39 items divided into four sections: demographics, diversity engagement [17], burnout [21], and perceived stress [22]. Sociodemographic domains include age, gender, sexual orientation, race, marital status, number of dependents, language, job title, and income.

### 2.2. Study measures

The primary objective of this study was to evaluate the correlation between burnout and diversity engagement, as well as the role of perceived stress in mediating this relationship (Supplementary Methods). To measure diversity engagement (predictor variable), we utilized the Diversity Engagement Survey (DES), a tool created and validated by the American Association of Medical Colleges and the University of Massachusetts that assesses progress in building engagement and inclusion [17]. The DES comprises 22 questions that evaluate three clusters: vision/purpose, camaraderie, and appreciation. Each cluster is further separated into domains providing a guide for interventions.

*Vision/purpose* provides a person with a compelling reason to achieve the goals of the institution. Vision/purpose clusters include domains of common purpose, access to opportunity, equitable reward and recognition, and cultural competence. *Camaraderie* gives people a sense of belonging and the ability to connect with others. The camaraderie cluster includes domains of trust and sense of belonging. *Appreciation* recognizes each individual’s contributions to the institution. Appreciation clusters include domains of appreciation of individual attributes and respect. A five-point Likert scale was used to quantify responses such as “strongly agree,” “agree”, “neither agree nor disagree,” “disagree,” and “strongly disagree.”

Mean DES scores were reported for the full cohort, per cluster and domain. We also calculated the overall favorable DES score by adding the percentage of participants responding with “strongly agree” or “agree” (Supplementary Table 1). Favorable and mean DES scores of our institution were compared to a benchmark sample that was calculated from 80,900 respondents from 50 institutions across the country [17]. Lower percentiles represent less engagement, while higher percentiles represent more engagement.

Burnout is a state of exhaustion that can increase mental distance from one’s job and a sense of reduced personal accomplishment [1–3]. We measure burnout (outcome variable) in study participants using a validated screening tool with two single-item questions regarding emotional exhaustion and depersonalization adapted from the Maslach Burnout Inventory [2,21]. This survey included a seven-point Likert scale where participants could rate the frequency of each item using “never (0),” “at least a few times a year (1),” “once a month or less (2),” “a few times a month (3),” “once a week (4),” “a few times a week (5),” and “every day (6).” Based on previously reported thresholds [21], each question was independently dichotomized as burned out if a respondent indicated “once a week” or more often. Therefore, for each question, a score cutoff >3 correlates with burnout [21]. Final scores ranged from 0, indicating low burnout, to 12, indicating high burnout.

Perceived stress, the mediator variable, was assessed using the validated Perceived Stress Scale (PSS-4), a widely used instrument with four questions that assesses participants’ feelings and perceptions of stress [22]. Since previous literature has found that shorter surveys are reliable and produce higher response and completion rates than longer surveys [23], the shorter version of the PSS was chosen, as opposed to the PSS-14 or PSS-10, to decrease the survey burden on participants. A five-point Likert scale was used to quantify participant responses to each statement: the choices were “very often,” “fairly often,” “sometimes,” “almost never,” and “never.” The final scores ranged from 0 to 16, with 0 and 16 correlating with low and high stress, respectively. While there are no specific cutoff scores for diagnosing stress, a previous study found that in a nationally representative sample, the average score was 4.2 (SD 2.8) for men and 4.7 (SD 3.1) for women [24].

### 2.3. Sample size and power calculation

No relevant data was available to conduct a power calculation prior to the study. Sample size was limited by the availability of willing participants in our health system. Statistical analysis was based on the total number of completed surveys.

### 2.4. Statistical analysis

Descriptive statistics were generated for demographic characteristics and reported as frequencies (percentages). Mean and standard deviation were calculated for the study measures. Mean DES scores were also calculated by occupation. K-means clustering based on mean DES scores by occupation was performed to identify groups of similar data points. We examined several criteria (cubic clustering criteria and pseudo-F statistic) to determine the optimal number of groups. We then calculated a mean score for the eight defined inclusion factors by occupation and created a plot of mean scores in ascending order by occupation for each factor. These plots provided a simple graphic method for visualizing the ability of DES to distinguish between occupations (Supplementary Fig. 1).

Unadjusted linear regression models were fit to test if diversity engagement measured by DES scores was associated with burnout and perceived stress scores. We also fitted unadjusted linear regression models to assess the strength of the association between demographic characteristics with burnout or perceived stress. We performed a mediation analysis with 5000 bootstrapped samples to evaluate whether perceived stress mediates the relationship between diversity engagement and burnout. Mediation analysis was additionally adjusted for a priori covariates including gender, marriage, race, income, sexual orientation, and occupation that were previously associated with burnout and perceived stress [4,8,12,25,26]. All tests were two-sided, and the significance level was set to 0.05. All statistical analyses were performed using SAS software version 9.4 [27].

## 3. Results

Of 683 eligible contacted participants, 176 were enrolled in the study, resulting in a 25.8% participation rate (*N* = 176/683). Only those who completed all the DES questions were included in the final analysis (*N* = 160). Most participants were female (58.5%), between the ages of 25 and 44 (62.4%), and identified as white (82.4%), heterosexual (86.9%), and married/living married (74.6%). There were 37.5% of the participants who worked as APPs, 28.4% as attending physicians, 20.5% as trainees (resident physicians or fellows), and 13.6% as other staff (Table 1). We observed an almost even distribution of years of experience, which ensures that the participants have been exposed to our work environment and therefore can accurately evaluate the level of diversity engagement (Supplementary Fig. 2).

### 3.1. Diversity engagement survey evaluation

**Fig. 1.**
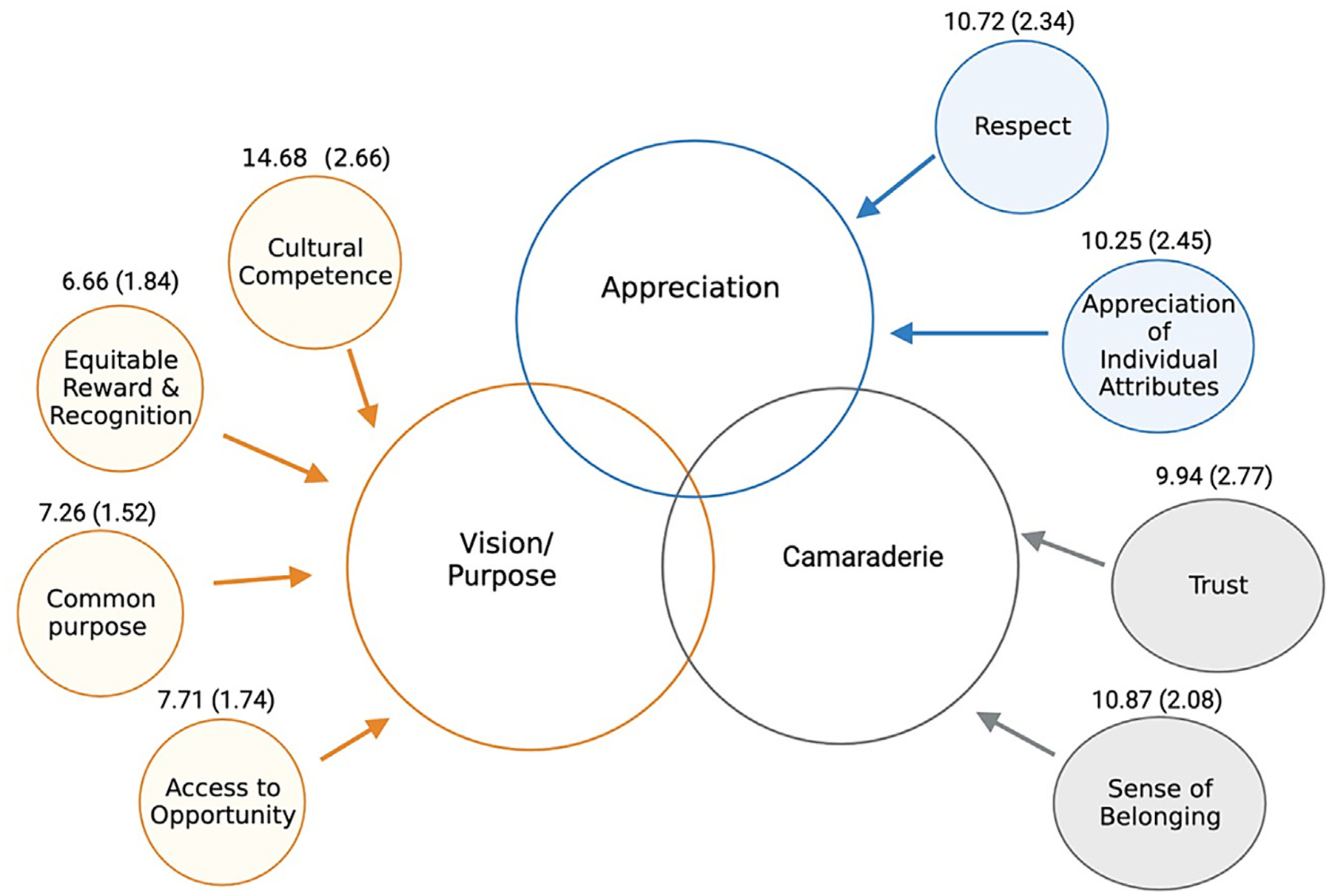
Diversity engagement clusters and the eight inclusion domains scores, with values expressed as mean (standard deviation). Common purpose, access to opportunity, and equitable reward scores ranged from 2 to 10 whereas cultural competence scores ranged from 4 to 10. Trust factor and sense of belonging scores ranged from 3 to 15. Appreciation of individual attributes and respect scores ranged from 3 to 15.

The mean DES score of our cohort was 78.3 (SD 14.3). Among the 160 participants the overall favorable score was 60%, which corresponds to the bottom third (33rd percentile) when compared to the national benchmark. When evaluating DES cluster factors, common purpose had a mean score of 7.3 (SD 1.5), access to opportunity 7.7 (SD 1.7), equitable reward and recognition 6.7 (SD 1.8), cultural competence 14.7 (SD 2.7), trust factor 9.9 (SD 2.8), sense of belonging 10.9 (SD 2.1), appreciation of individual attributes 10.3 (SD 2.5) and respect 10.7 (SD 2.3) (Fig. 1).

**Fig. 2.**
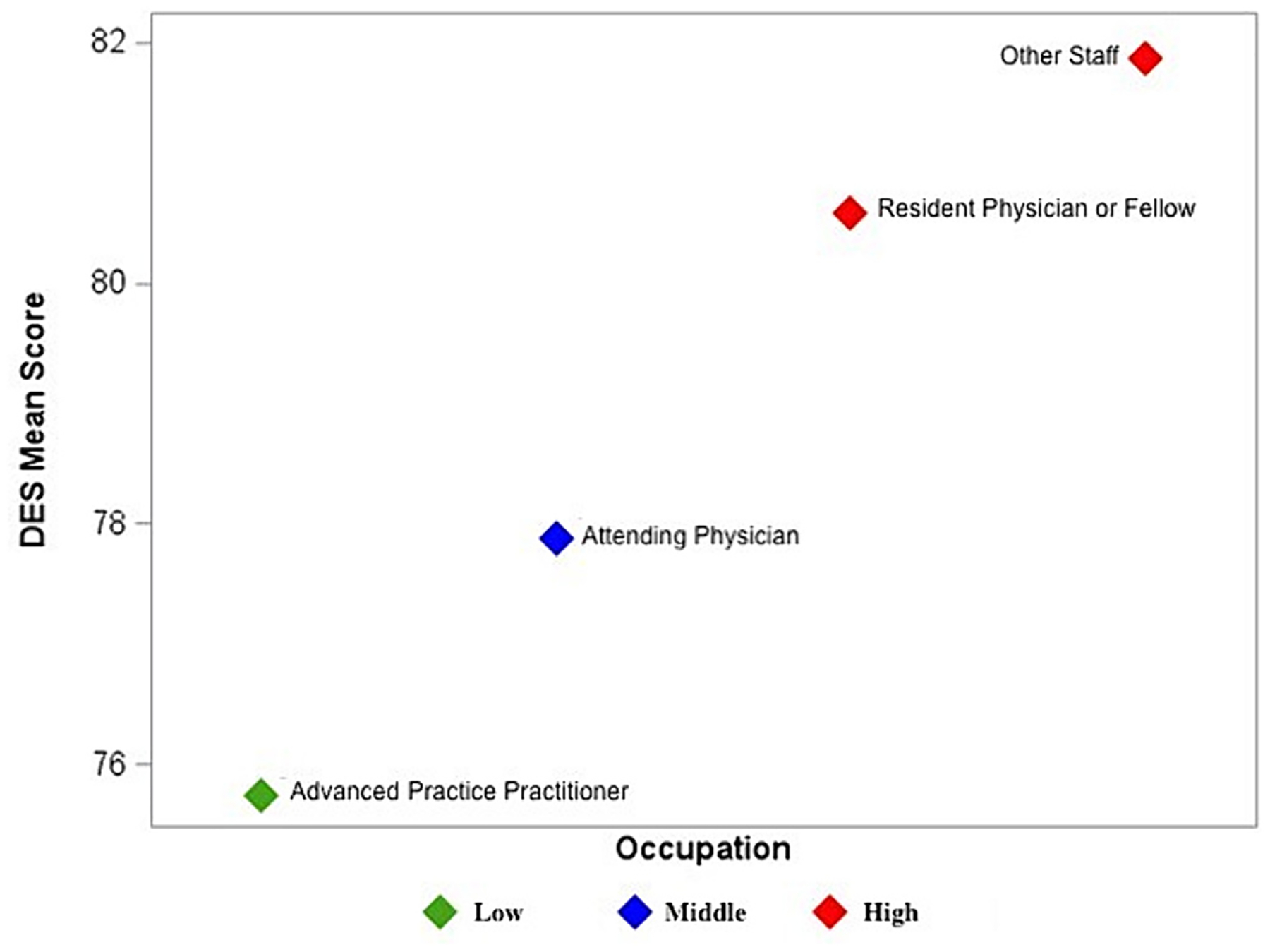
K-means cluster analysis based on mean Diversity Engagement Survey (DES) scores for each occupation. Colors indicate distinct clusters for low, middle, and high mean DES score. High DES score represents more perceived diversity engagement, while low DES score represents less perceived diversity engagement.

When analyzing mean DES scores by occupation, other staff had the highest score of 81.9 (SD 13.4) whereas APPs had the lowest of 75.7 (SD 15.6) (Fig. 2). When evaluating DES cluster factors for each occupation against a national benchmark [17], resident physicians/fellows scored in the lowest 25th percentile for common purpose, cultural competence, trust, appreciation, and respect. Attendings scored in the 50th percentile for most of the cluster factors except for common purpose, trust, and respect, in which they scored in the 25th percentile. Similarly, APPs scored in the 50th percentile for all cluster factors except trust and respect, in which they scored in the 25th percentile. Other staff scored in the 50th percentile for every cluster factor (Supplementary Fig. 1).

### 3.2. Burnout and perceived stress evaluation

Overall, the mean scores were 4.4 (SD 3.2) for burnout and 4.8 (SD 2.6) for perceived stress. Results showed that races other than white had lower burnout when compared to white participants (β = −1.53 [95% CI −2.76, −0.30], *P* = 0.015). Compared to those who identified as straight, people who identified as lesbian, gay or bisexual (β = 0.06 [95% CI −1.45, 1.56], *P* = 0.040) showed an association with higher perceived stress scores. Similarly, those with a salary ≤$53,700 had higher perceived stress (β = 3.14 [95% CI 1.33, 4.95], *P* = 0.002) compared to the highest earners ≥$163,30. The number of dependents did not have any association with burnout (*P* = 0.637) or perceived stress (*P* = 0.280) (Table 2). Characteristics associated with lower perceived stress were male gender (*P* = 0.007) and married/living like married (*P* = 0.041).

### 3.3. Mediation analysis

**Fig. 3.**
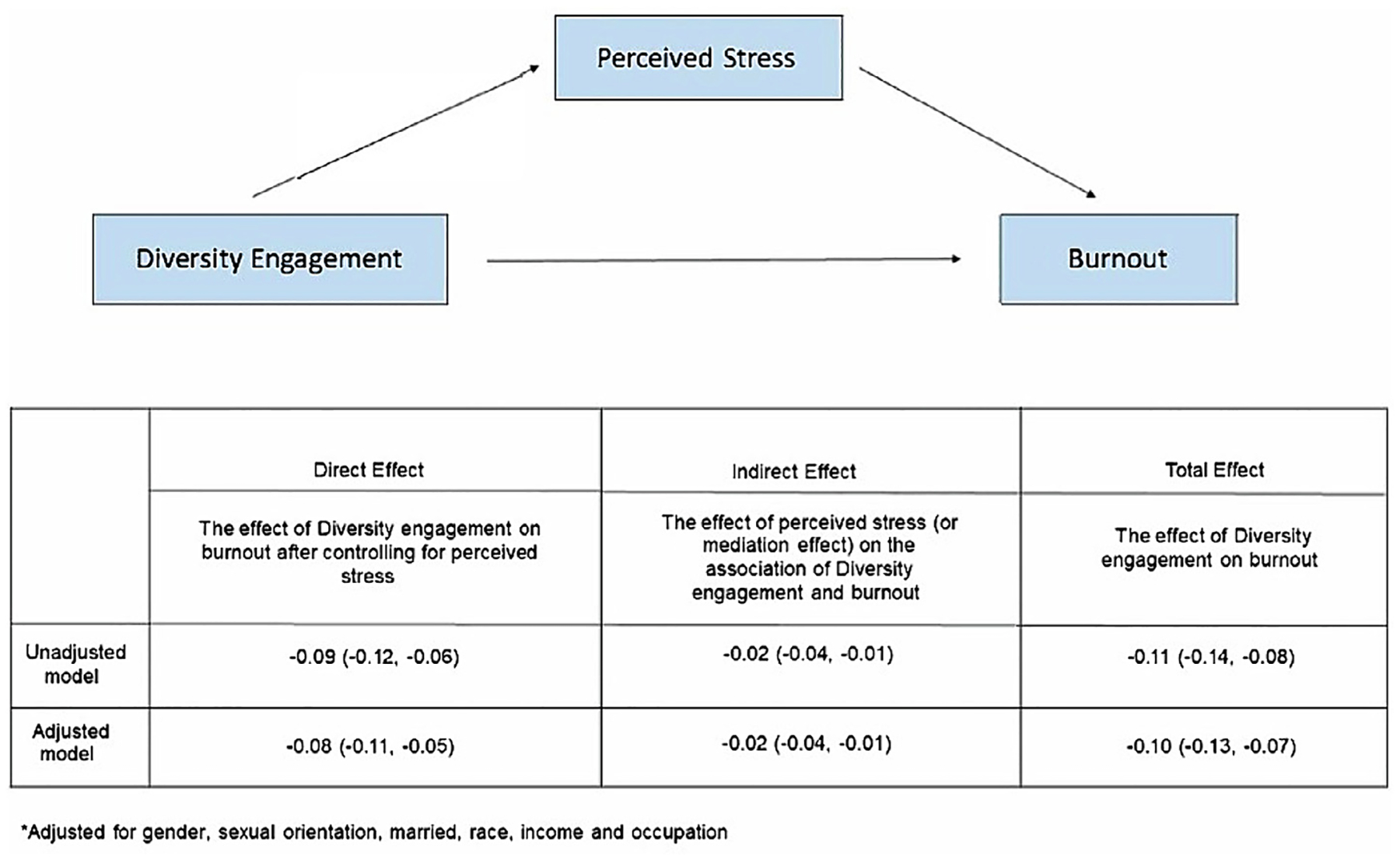
Perceived stress partially mediates the association between diversity engagement and burnout. Model was adjusted for covariates including male gender, married, race, income, sexual orientation, and occupation.

We observed that higher DES scores predicted lower burnout (β = −0.11 [95% CI −0.14, −0.08], *P* < 0.001) and lower perceived stress (β = −0.05 [95% CI −0.08, −0.03], P < 0.001). Higher perceived stress scores were associated with higher burnout (β = 0.56 [95% CI 0.39, 0.72], P < 0.001). After accounting for a priori covariates, DES scores had a significant direct effect on burnout (β = −0.08; *P* < 0.0001), such that higher DES scores were significantly associated with lower burnout scores. Mediation analyses estimated that the total effect on burnout was β = −0.10, P < 0.0001, which comprises the direct effect of diversity engagement (β = −0.08, *p* < 0.0001) and indirect effect of perceived stress (β = −0.02, *p* = 0.0048) (Fig. 3). This mediation analysis showed that approximately 22% of the total effect can be explained through the perceived stress mediation pathway.

## 4. Discussion

Our survey-based study across a multi-site US-based hospital system demonstrated that higher diversity engagement was associated with reduced burnout and perceived stress. Because burnout has been found to disproportionally affect anesthesia providers compared to clinicians in other specialties [4,5], our findings highlight the potential for interventions focused on improving diversity engagement to reduce burnout by implementing top-down strategies driven by department leaders.

Diversity engagement, measured through the DES, revealed a 33rd percentile favorable score (60%), indicating weak perceived engagement and inclusion by participants. When examining specific factors such as common purpose, trust, and respect scores, we observed those were in the lowest 33rd percentile. When broken down by occupation, attendings, residents, and APPs scored in the lowest 25th percentile in those factors, specifically for trust and respect (Supplementary Fig. 1). These low scores may suggest a sense of disconnection from the organization’s mission, distrust in policies, practices, and procedures, or feeling as though the environment does not welcome diverse perspectives [17]. Therefore, we propose that diversity engagement may contribute to burnout particularly via low common purpose, trust, and respect.

A way to improve burnout may require incorporating diversity goals into leadership succession planning. Perceived leadership quality significantly influences well-being and workplace culture [11,28,29]. A prior study found that physician burnout and satisfaction levels are strongly associated with perceived leadership quality. Participants were asked to evaluate their leader using a nine-item index that measured leadership behaviors. These scores were then summed to compute a composite leadership score. Researchers found that each one-point increase in composite leadership score was associated with a 3.3% decrease in the likelihood of burnout of the physicians supervised [29]. This suggests that interventions focused on improving leadership opportunities and skills, such as empowering team members, informing team members, soliciting their input, providing feedback, and nurturing professional development, may reduce burnout and increase job satisfaction. Another study showed that adopting the modified version of the 2013 Mayo Clinic intervention, using the Listen-Act-Develop model can reduce burnout and engage physicians in the organization’s mission [30]. Based on previous literature, preventative burnout factors were grouped into 7 dimensions: workload, efficiency, flexibility/control over work, work-life integration, alignment of individual and organizational values, social support/community at work, and the degree of meaning derived from work [30]. After interventions specific to each work unit were implemented, all seven Mayo Clinic work units saw a median change of 11% in absolute reduction of burnout, and five clinics had an 8% improvement in satisfaction. Thus, we propose a similarly structured approach that includes interventions aimed at improving leadership skills and diversifying leadership roles.

Occupational stress is also associated with burnout [11,14,31]; however, in our study it only explained 22% of the relationship. When comparing our findings to those from a prior study that used a nationally representative sample, we observed that our mean PSS-4 score of 4.8 (SD 2.6) was similar to the mean PSS-4 score of 4.2 (SD 2.8) for men and 4.7 (SD 3.1) for women in that study [24]. The comparable PSS-4 scores between our study and the national population are also similar to those from a study conducted on Belgian anesthesiologists, which found only moderate levels of stress that were no higher than those of the general population [31]. This contrasts with results from some previous studies on anesthesiology residents, one of which found 67.5% of residents to have stress levels above the national average, and 41.25% to be severely stressed [32]. Our study findings suggest that stress alone may not completely explain the increased risk of burnout among anesthesia providers and that other factors need to be explored.

Our study has inherent limitations. Our cohort did not reflect the percentage of racial and ethnic groups working in anesthesiology [33]. Additionally, the cohort was primarily wealthy and heterosexual. This may have compromised our ability to assess how sociodemographic factors influence burnout and perceived stress. Despite the limited diversity of our cohort, the results indicate that diversity engagement is advantageous and beneficial because it is correlated with reduced burnout. Although the survey was disbursed at multiple community and academic hospital locations, our data only represents a single healthcare system and geographical area, potentially limiting external validity. Our study did not collect data on hours of work per week, which may be a quantitative factor associated with burnout and stress. The PSS-4, as noted by the original authors, has decreased internal reliability, so it may not measure perceived stress as accurately as the PSS-10 or PSS-14 [22].

We recognize that the “Others” occupation group captures a heterogeneous group of employees (i.e. anesthesia technicians and other staff). However, we decided to include them in our analysis to measure the diversity engagement of all department members. Although physicians and other staff differ in many clinical aspects, our results highlight the difference in diversity engagement between them and may serve as the foundation for future studies to disentangle the factors contributing to these differences. Lastly, our study is cross-sectional and prevents the assessment of changes over time. Future studies should consider a longitudinal administration of the DES and burnout surveys after an intervention to determine if diversity engagement strategies lower burnout.

This cross-sectional survey study demonstrates a correlation between diversity engagement and burnout, with perceived stress partially mediating this relationship. Our findings suggest an important role of diversity engagement in fostering a healthier working environment. These associations provide a foundation for future interventions aimed at cultivating a more inclusive work environment in anesthesiology departments.

## Supplementary Material

Sup table 1

Sup Fig 1

## Figures and Tables

**Table 1:** Demographic characteristics of the enrolled participants (*N* = 176).

Primary Cohort	
Characteristic	Frequency (%)
**Age, years** ^a^	
≤24	4 (2.3)
25–44	108 (62.4)
≥45	61(35.3)
**Gender** ^b^	
Female	103 (58.5)
Male	73 (41.5)
**Sexual orientation**	
Straight	152 (86.9)
Lesbian or gay, bisexual	12 (6.9)
Other	11 (6.3)
**Race**	
White	145 (82.4)
Other^c^	31 (17.6)
**English as primary language**	164 (94.8)
**Married/living like married**	129 (74.6)
**Number of dependents**	
None	84 (48.8)
One or More	88 (51.2)
**Primary caretaker status**	58 (33.3)
**Occupation**	
Attending physician	50 (28.4)
Trainee (resident or fellow)	36 (20.5)
Advanced practice provider^d^	66 (37.5)
Other staff^e^	24 (13.6)
**Academic Hospitals** ^f^	143 (81.3)
**Income**	
≤$53,700	8 (4.7)
$53,701–$85,500	25 (14.8)
$85,501–$163,300	21 (12.4)
≥$163,301	115 (68.1)

a Categorized based on the 2020 census.

b Defined as an individual’s subjective sense of oneself as a gendered person. Participants self-identified as female, male, or other.

c Includes Asian, Middle Eastern or Northern African, Black or Latino.

d Defined as certified registered nurse anesthetists, nurse practitioners, and physician assistants.

e Defined as anesthesiology technicians and other staff.

f Defined as a tertiary hospital affiliated with a medical school and/or residency program.

**Table 2:** Univariate associations between demographic characteristics and study measures (*N* = 176).

	Burnout		Perceived Stress	
Characteristic	Coefficient (95% CI^a^)	*p*-value	Coefficient (95% CI)	p-value
**Age**		0.801		0.794
≤24	0.49 (−2.78, 3.75)		−0.47 (−3.11, 2.17)	
25–44	0.33 (−0.68, 1.34)		−0.27 (−1.09, 0.55)	
≥45	Reference		Reference	
**Male gender (vs. Female)**	−0.81 (−1.77, 0.15)	0.097	−1.05 (−1.81, −0.28)	0.007
**Sexual orientation**		0.131		0.040
Lesbian or gay, bisexual	0.92 (−0.97, 2.80)		0.06 (−1.45, 1.56)	
Other	1.84 (−0.12, 3.81)		2.03 (0.46, 3.59)	
Straight	Reference		Reference	
**Race**				
Other	−1.53 (−2.76, −0.30)	0.015	0.45 (−0.56, 1.46)	0.381
White	Reference		Reference	
**Married/living like married**	−0.78 (−1.88, 0.31)	0.161	−0.92 (−1.81, −0.04)	0.041
**Number of dependents**		0.637		0.280
One or more	−0.23 (−1.20, 0.74)		−0.43 (−1.20, 0.35)	
None	Reference		Reference	
**Primary caretaker status**		0.641		0.559
Yes	−0.24 (−1.26, 0.78)		0.24 (−0.58, 1.07)	
No	Reference		Reference	
**Occupation**		0.515		0.081
Attending physician	−0.35 (−1.92, 1.22)		−1.29 (−2.54, −0.04)	
Trainee	0.56 (−1.11, 2.22)		−0.46 (−1.78, 0.87)	
Advanced provider	0.42 (−1.09, 1.92)		−1.31 (−2.51, −0.11)	
Other staff	Reference		Reference	
**Income**		0.628		0.002
≤$53,700	0.31 (−1.99, 2.61)		3.14 (1.33, 4.95)	
$53,701–$85,500	−0.21 (−1.59, 1.18)		1.08 (−0.01, 2.17)	
$85,501–$163,300	−0.95 (−2.44, 0.54)		0.74 (−0.44, 1.91)	
≥$163,301	Reference		Reference	

a CI = confidence interval; p-value < 0.05 indicates statistical significance.

## Data Availability

Data will be made available on request.

## Appendix A. Supplementary data

Supplementary data to this article can be found online at https://doi.org/10.1016/j.jcadva.2024.100027.
