# Supplementary material for: Diversity engagement is associated with lower burnout among anesthesia providers
Source: JCA Adv. Author manuscript; Available in PMC 2026 Jan 17. (PMC12810883; doi:10.1016/j.jcadva.2024.100027)
Supplement: Sup table 1 [file NIHMS2134689-supplement-Sup_table_1.docx]

**Supplementary Figure 1.** Mean cluster factor scores and occupation cluster membership (statistical group) based on engagement.


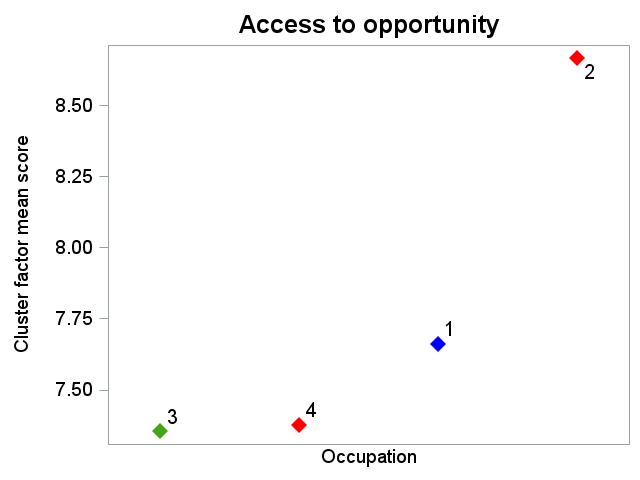


Resident Physician or Fellow

Attending Physician

Advanced Practice

Other Staff

Practitioner


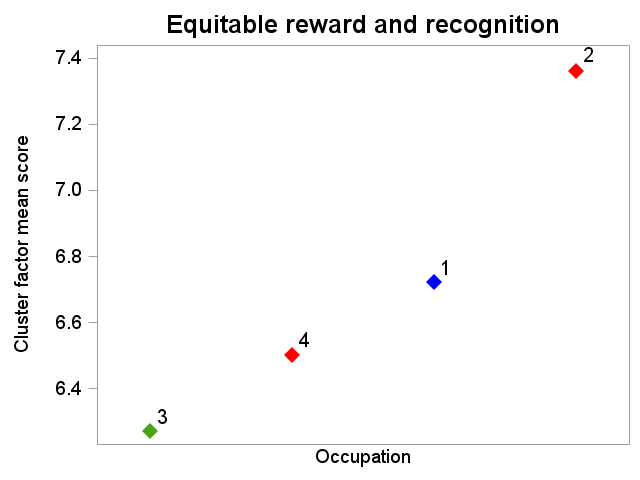


Resident Physician or Fellow

Attending Physician

Advanced Practice Practitioner

Other Staff


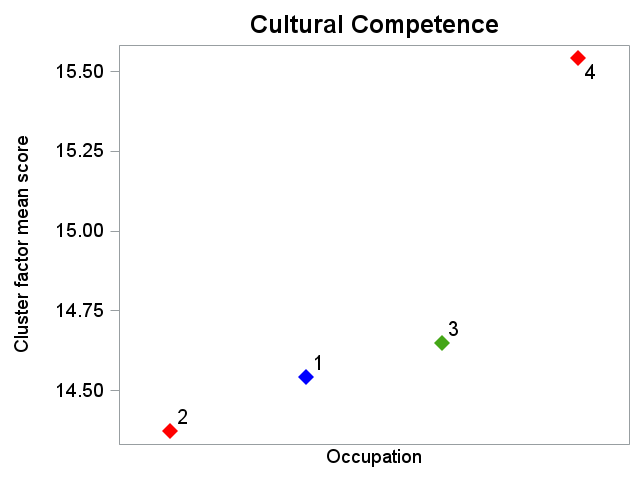


Other Staff

Attending Physician

Advanced Practice

Practitioner

Resident Physician or Fellow


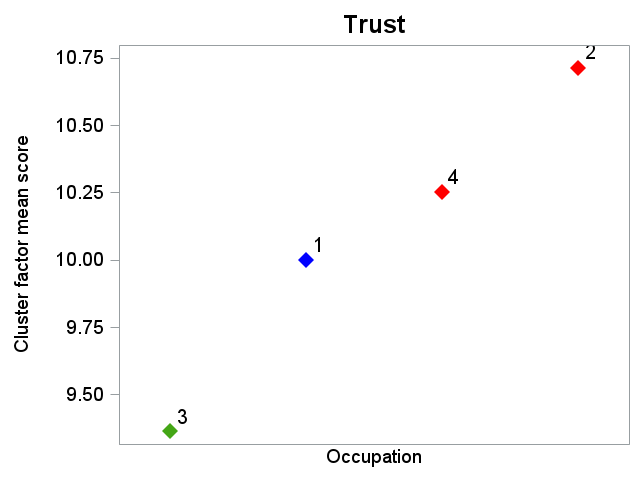


Other Staff

Attending Physician

Advanced Practice Practitioner

Resident Physician or Fellow


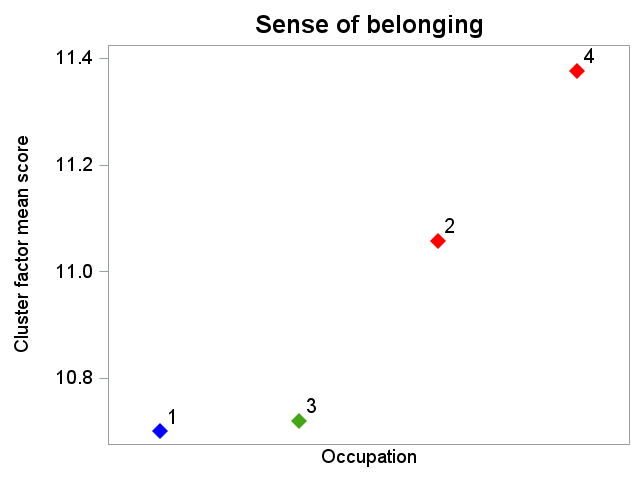


Other Staff

Resident Physician

Attending Physician

Advanced Practice Practitioner

or Fellow


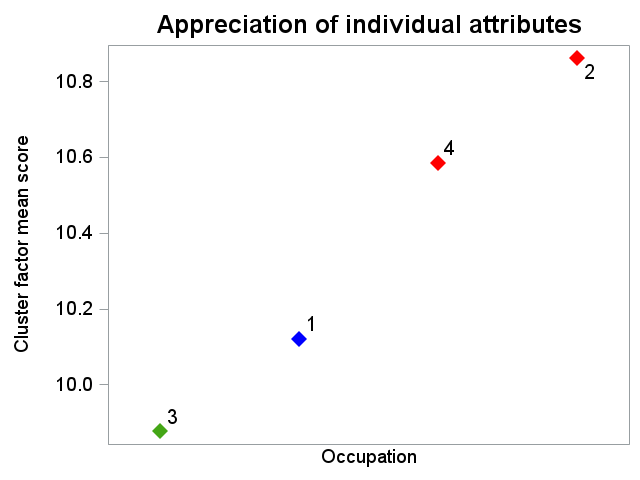


Other Staff

Attending Physician

Resident Physician or Fellow

Advanced Practice Practitioner


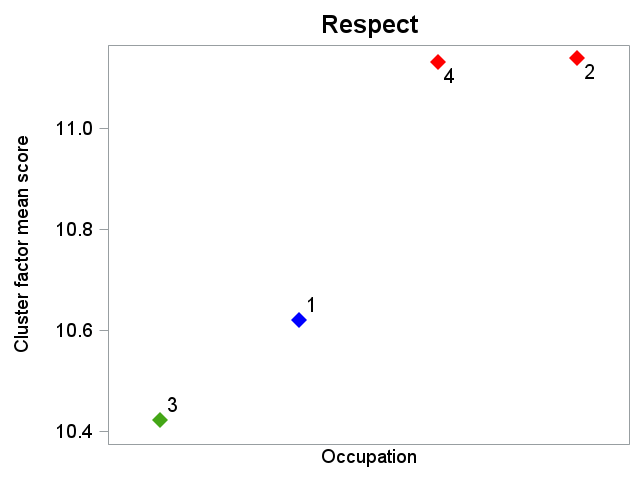


Other Staff

Attending Physician

Advanced Practice Practitioner

or Fellow

Resident Physician

Mean cluster factor scores and occupation cluster membership (statistical group) based on engagement. Each point represents an occupation. K-means cluster analysis was based on each occupation group mean DES score. Cluster 1 (green), 2 (blue), and 3 (red) refer to the statistical group based on low, middle and high mean DES score, respectively.

Supplementary Figure 2. Participant years of service at the Institution
