# Supplementary material for: Diversity engagement is associated with lower burnout among anesthesia providers
Source: JCA Adv. Author manuscript; Available in PMC 2026 Jan 17. (PMC12810883; doi:10.1016/j.jcadva.2024.100027)
Supplement: Sup Fig 1 [file NIHMS2134689-supplement-Sup_Fig_1.docx]

Supplementary Table 1: Favorable Score Calculations

| Survey Question Number | Strongly Agree (%) | Agree (%) | Favorable Score |
| --- | --- | --- | --- |
| Q1 | 8% | 32% | 40% |
| Q2 | 12% | 38% | 50% |
| Q3 | 6% | 30% | 36% |
| Q4 | 19% | 60% | 79% |
| Q5 | 23% | 49% | 73% |
| Q6 | 4% | 28% | 32% |
| Q7 | 22% | 56% | 78% |
| Q8 | 27% | 43% | 70% |
| Q9 | 28% | 47% | 74% |
| Q10 | 13% | 46% | 59% |
| Q11 | 9% | 39% | 48% |
| Q12 | 18% | 56% | 74% |
| Q13 | 13% | 40% | 53% |
| Q14 | 34% | 51% | 85% |
| Q15 | 13% | 49% | 62% |
| Q16 | 7% | 41% | 48% |
| Q17 | 8% | 42% | 50% |
| Q18 | 11% | 49% | 60% |
| Q19 | 16% | 46% | 63% |
| Q20 | 13% | 51% | 64% |
| Q21 | 23% | 54% | 76% |
| Q22 | 11% | 47% | 58% |
|  | Favorable Score of Study = 60% | | |
